# Quantitative assessment of the blood-brain barrier opening caused by *Streptococcus agalactiae* hyaluronidase in a BALB/c mouse model

**DOI:** 10.1038/s41598-017-13234-1

**Published:** 2017-10-19

**Authors:** Su Luo, Qing Cao, Ke Ma, Zhaofei Wang, Guangjin Liu, Chengping Lu, Yongjie Liu

**Affiliations:** College of Veterinary Medicine, Nanjing Agricultural University, Nanjing, 210095 China

## Abstract

*Streptococcus agalactiae* is a pathogen causing meningitis in animals and humans. However, little is known about the entry of *S. agalactiae* into brain tissue. In this study, we developed a BALB/c mouse model based on the intravenous injection of β-galactosidase-positive *Escherichia coli* M5 as an indicator of blood-brain barrier (BBB) opening. Under physiological conditions, the BBB is impermeable to *E. coli* M5. In pathological conditions caused by *S. agalactiae*, *E. coli* M5 is capable of penetrating the brain through a disrupted BBB. The level of BBB opening can be assessed by quantitative measurement of *E. coli* M5 loads per gram of brain tissue. Further, we used the model to evaluate the role of *S. agalactiae* hyaluronidase in BBB opening. The inactivation of *hylB* gene encoding a hyaluronidase, HylB, resulted in significantly decreased *E. coli* M5 colonization, and the intravenous injection of purified HylB protein induced BBB opening in a dose-dependent manner. This finding verified the direct role of HylB in BBB invasion and traversal, and further demonstrated the practicability of the *in vivo* mouse model established in this study. This model will help to understand the *S. agalactiae*–host interactions that are involved in this bacterial traversal of the BBB and to develop efficacious strategies to prevent central nervous system infections.

## Introduction

*Streptococcus agalactiae*, also known as Group B *Streptococcus* (GBS), is a Gram-positive, opportunistic pathogen that colonizes the gastrointestinal and genitourinary tracts of up to 50% of healthy adults^1^. It also causes septicemia, meningitis and pneumonia in neonates and is the main reason for significant morbidity in pregnant women, the elderly and immunocompromised adults^2–4^. *S. agalactiae* can also infect the mammary glands of cows, where it can survive for a long period of time, causing clinical or subclinical mastitis^5^. In addition, in recent years, it has been reported that *S. agalactiae* can cause meningoencephalitis in fish and bring losses to aquaculture^6^. Numerous outbreaks of *S. agalactiae* infections have been described in multiple fish farms, especially tilapia farms^7–9^. Since 2009, an outbreak of severe infectious GBS disease has occurred in tilapia farms in the south of China, causing tremendous economic losses due to high mortality in the infected fish^10^.

The pathogenesis of meningitis caused by *S. agalactiae* has not been fully elucidated. It is well known that only pathogens entering the central nervous system (CNS) can cause meningitis. To gain access into the CNS, *S. agalactiae* must cross the blood-brain barrier (BBB), which is primarily comprised of a single layer of specialized brain microvascular endothelial cells (BMECs)^11,12^. This unique brain endothelial physiological barrier seals the CNS^13–15^, and regulates passage of molecules, nutrients, and infectious agents into the brain^16^. Pathogens can cross the BBB transcellularly, paracellularly and by a “Trojan-horse” mechanism^17^. Some studies have demonstrated that *S. agalactiae* virulence factors contribute to the adherence and invasion of human BMECs (hBMECs), resulting in the activation of acute inflammatory responses that inevitably disrupt the integrity of the BBB. For example, the well-known virulence factor β-hemolysin/cytolysin stimulated the expression of genes linked to neutrophil recruitment and activation, such as IL-8 and ICAM-1, which act to facilitate neutrophil migration across polar hBMEC monolayers^18^. PilA, the pilus tip adhesin of GBS, had capability in promoting the expression of neutrophil chemokines IL-8, CXCL-1, CXCL-2, CCL-20 and IL-6 in brain endothelium, and therefore increased the permeability of the BBB^19^. Several investigations in animals have also shown that GBS can penetrate the CNS by crossing the BBB after a prolonged period of bacteremia^20,21^. Therefore, the BBB plays an important role in controlling the entry of pathogens into the brain.

Increased permeability of the BBB can be seen in bacterial meningitis caused by *S. agalactiae* ^22,23^. However, how this bacterium crosses the BBB and enters the CNS is not clearly understood. Therefore, it is of crucial importance to characterize BBB permeability in order to better understand the pathogenesis of meningitis. In our previous study, the inactivation of *hylB* gene encoding a hyaluronidase, HylB, caused the significantly decreased brain bacterial counts in mice^24^. However, whether HylB directly acts on the BBB opening remains unclear. In this study, we sought to establish a model to evaluate BBB opening by screening a bacterial strain as an indicator, and used this model to quantitatively evaluate the direct role of HylB in *S. agalactiae* penetration across BBB.

## Results

### The screening of an indicator strain

**Figure 1:**
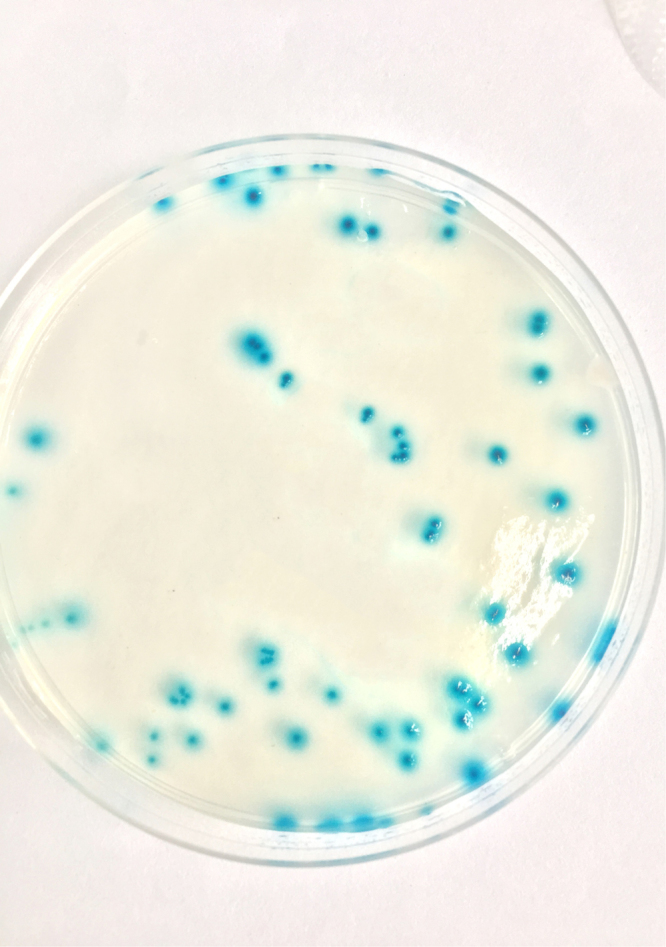
The growth of *E. coli* M5 on an M63 plate. The bacteria formed blue colonies on M63 medium containing X-Gal.

To make the counting easy, we aimed to find a bacterium with a colony morphology distinct from that of *S. agalactiae*. The results showed that among the M1 to M5 *E. coli* mutants, only the M5 strain generated characteristic blue clones on M63 media (Fig. 1), indicating that it was β- galactosidase-positive.

### Determination of *E. coli* M5 virulence in mice

To determine whether *E. coli* M5 was virulent to mice, we performed bacterial infection in BALB/c mice. Interestingly, it was observed that none of the mice infected with 2 × 10^9^ CFU of *E. coli* M5 showed any signs of illness, and there was zero mortality throughout the experimental period of 7 d. The diet and mental state of the experimental mice were identical to those of the control group. The result indicated that *E. coli* M5 was avirulent to mice.

### Kinetics of *E. coli* presence in blood of mice

**Figure 2:**
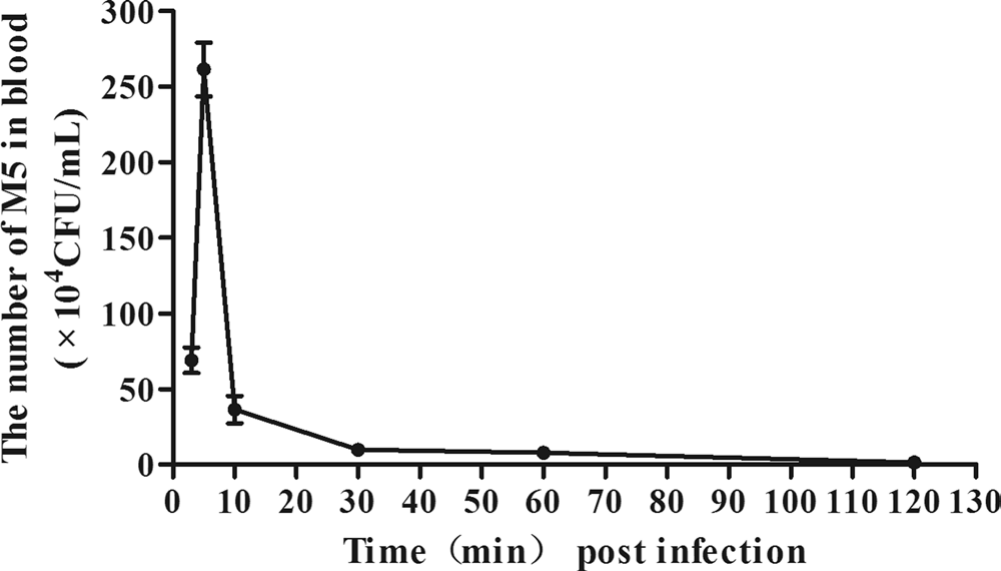
Metabolism of *E. coli* M5 in mice. The recovered bacteria are expressed as CFU per milliliter of blood.

To investigate the rate of *E. coli* M5 metabolism in mice, the mice were injected intravenously with M5, and blood samples were collected from each mouse at intervals of 3 min, 5 min, 10 min, 30 min, 60 min and 120 min post-infection. The CFU enumeration results showed that the number of M5 cells in the blood increased from 6 × 10^5^ CFU/mL at 3 min to a peak value of approximately 2.5 × 10^6^ CFU/mL at 5 min and then decreased dramatically to 3 × 10^5^ CFU/mL at 10 min. After one hour, bacteria could hardly be detected, and they had clearly been removed from circulation within 2 hours after intravenous injection (Fig. 2). No bacteria were detected in the brain at any time points.

### Determination of a challenge concentration of *S. agalactiae*

**Figure 3:**
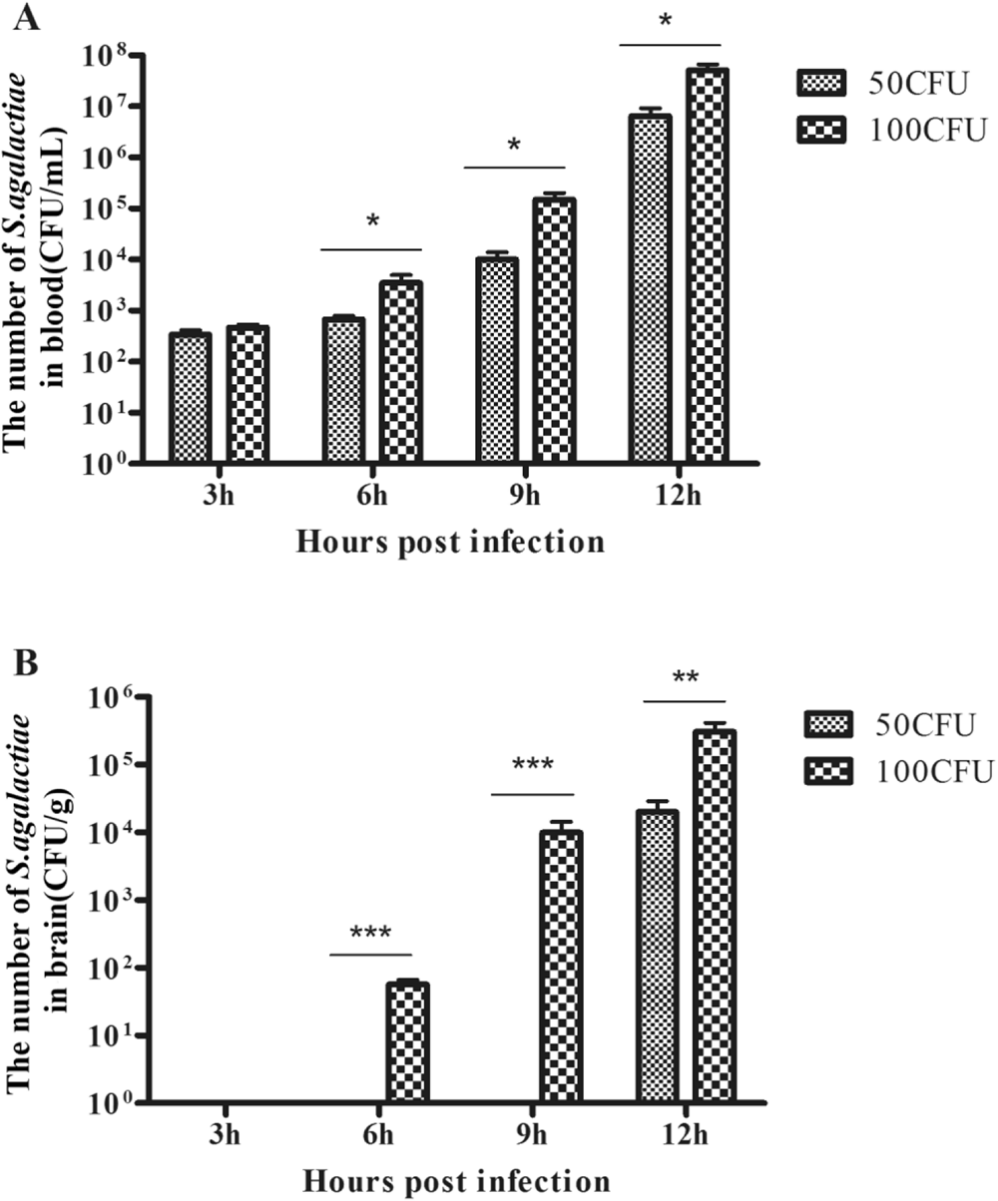
Detection of *S. agalactiae* in blood and the brain tissues. (**A**) Bacterial loads in the blood; (**B**) bacterial loads in the brain. Groups of four BALB/c mice were inoculated with 50 CFU or 100 CFU of *S. agalactiae* and killed at different time points post-infection. The CFU of *S. agalactiae* in the blood and the brain were quantified and described as the mean and S.D. *P < 0.05, **P < 0.01 or ***P < 0.001 indicates significantly different bacterial loads between the two infection groups.

We first sought to determine whether the *S. agalactiae* GD201008-001 strain causes significant BBB permeability by intraperitoneal infection. It is important to determine an appropriate initial inoculation concentration for *S. agalactiae* to create an *in vivo* model. We chose values of 5-fold (50 CFU) and 10-fold (100 CFU) that of the median lethal dose (LD_50 < _10 CFU)^25^ of *S. agalactiae* to inoculate the mice. The result showed that with the duration of infection, the number of GD201008-001 cells increased in both the blood (Fig. 3A) and the brain (Fig. 3B), suggesting that GD201008-001 was capable of replicating within the bloodstream and spreading to the brain. GD201008-001 began to appear in the blood at 3 h post-infection, 3 h earlier than in the brain. The bacteria could be detected in the brain at 6 h post-infection when we injected 100 CFU of *S. agalactiae*. However, we could not detect any bacteria until 12 h post-infection in the 50 CFU group. This result suggests that the intraperitoneal infection with 100 CFU of *S. agalactiae* is more appropriate to induce BBB opening in this mouse model.

### Evaluation of BBB opening

**Figure 4:**
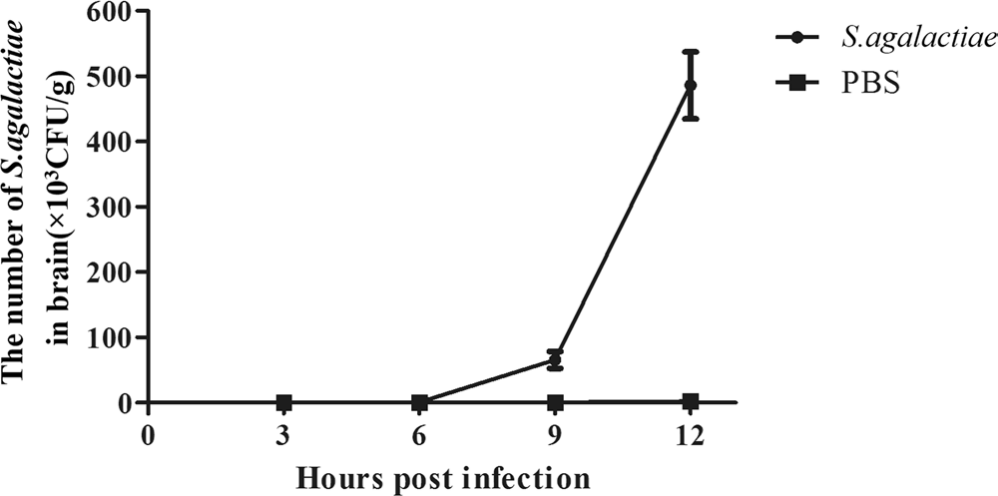
The changes in *S. agalactiae* loads in brain tissues of mice over time. The recovered *S. agalactiae* are expressed as CFU per gram of brain.

**Figure 5:**
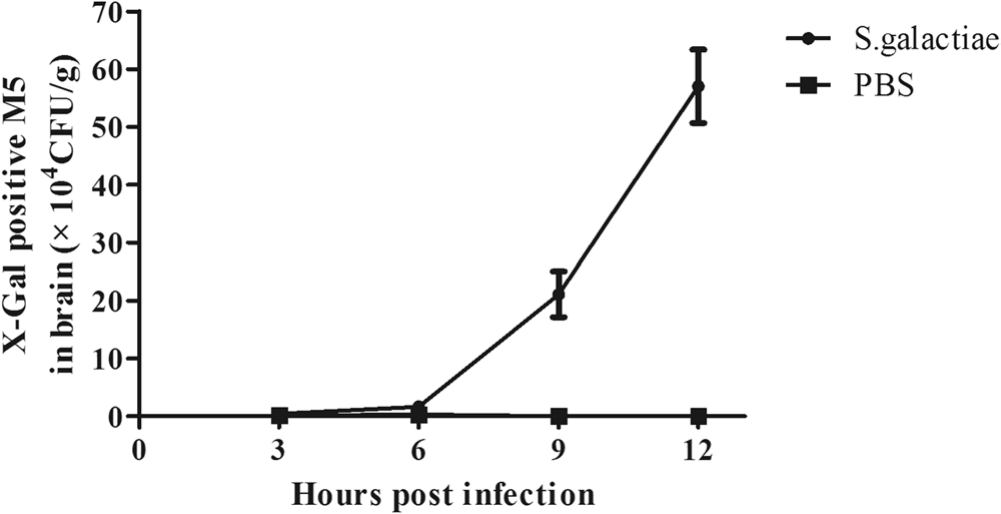
Evaluation of BBB opening caused by *S. agalactiae*. Mice were intraperitoneally infected with *S. agalactiae*, and the X-Gal-positive M5 were given by intravenous injection at different time points post infection. Five minutes later, the mice were killed, and the brains were removed for quantification of M5. The data are expressed as CFU per gram of brain.

Groups of five mice were challenged with 100 CFU of GD201008-001. At 3 h, 6 h, 9 h and 12 h after challenge, the indicator strain *E. coli* M5 was inoculated intravenously into each group. In determining the sampling time points, based on Fig. 2, more M5 tracers were detected in the blood at 5 min. Therefore, 5 min after injection was selected to quantify the degree of BBB permeability. Our data showed that GD201008-001 began to appear in the brain 6 h post-infection, and with the duration of infection, the bacterial number increased (Fig. 4). M5 could be detected 3 h after *S. agalactiae* inoculation, and the number of M5 had a similar increasing trend as GD201008-001 (Fig. 5). This result suggests that *E. coli* M5 may be used as an appropriate indicator for describing the degree of BBB opening.

### Detection of BBB opening caused by *S. agalactiae* hyaluronidase

**Figure 6:**
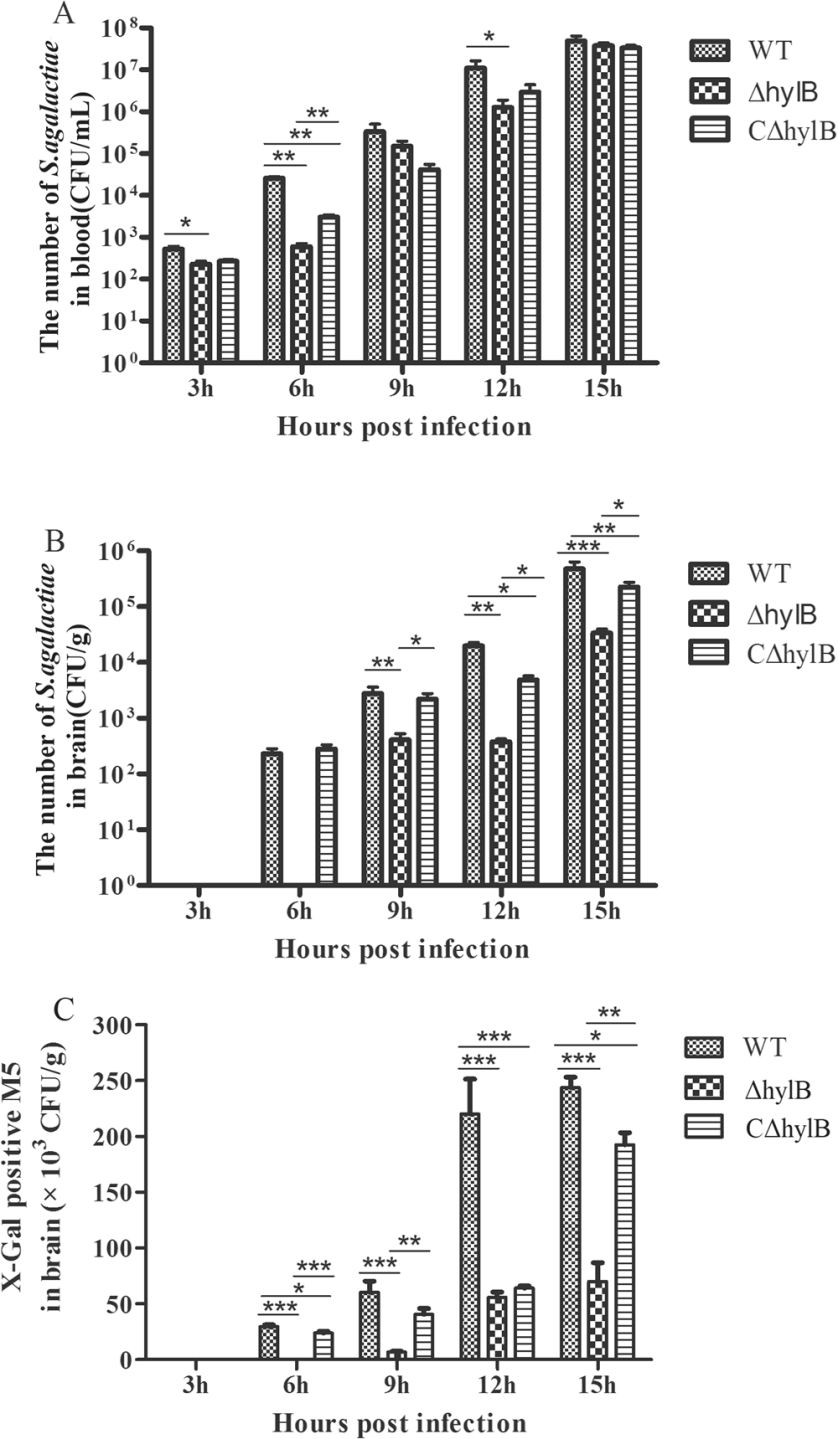
Evaluation of BBB opening in mice infected intraperitoneally with the *S. agalactiae* wild-type (WT), mutant (Δ*hylB*), and complemented (CΔ*hylB*) strains. Groups of 40 BALB/c mice were inoculated with 100 CFU *S. agalactiae* and its derivatives. At 3 h, 6 h, 9 h, 12 h, and 15 h post infection, the blood (**A**) and brain (**B**) tissues were collected from four mice of each groups for *S. agalactiae* quantification. Meanwhile, at each time point, another four mice from each group were injected intravenously with *E. coli* M5 (2 × 10^8^ CFU). Five minutes later, the mice were killed, and the brains were removed for quantification of M5 (**C**). Bacterial loads in the blood are expressed as CFU/mL, and those in the brains are expressed as CFU/g of tissue. *P < 0.05, **P < 0.01 or ***P < 0.001 indicates significantly different bacterial loads between the two infection groups.

Previous study showed that hyaluronidase contributed to *S. agalactiae* penetration into the CNS^24^. To verify the utility of this mouse model, the mice were infected with the wild-type *S. agalactiae*, Δ*hylB* and CΔ*hylB* strains, and then *E. coli* M5 was administered to the mice. After intraperitoneal infection with 100 CFU of *S. agalactiae* and its derivatives, the numbers of the three bacterial strains in the blood increased rapidly from <10^3^ CFU/mL at 3 h to >10^7^ CFU/mL at 15 h (Fig. 6A). In the brains, the wild-type and the complemented strain CΔ*hylB* were present 6 h post-infection, which was earlier than the mutant Δ*hylB* (Fig. 6B). Compared with the wild-type and complemented strains, for the *hylB* mutant, the numbers were lower at each time point in the brains. As time passed, the number of bacteria in the brains increased to >10^5^ CFU/g at 15 h post-infection. *E. coli* M5 in the wild-type and CΔ*hylB* groups could be detected in the brain tissues 6 h post-infection, and the increasing trend of the number of M5 cells was similar to that of *S. agalactiae* (Fig. 6C). The sudden increase in the number of M5 at 15 h indicated that the BBB of the mice had opened to a great degree. However, in the Δ*hylB* group, the Δ*hylB* strain and M5 were not detected until 9 h post-infection, and the amount of M5 was significantly lower than that of the wild-type group at 9 h post-infection (*P* < 0.001). At 12 h and 15 h, the difference in the number of M5 cells between the wild-type and Δ*hylB* groups reached a highly significant difference (*P* < 0.001). Although the number of M5 cells in the CΔ*hylB* group was lower than that in the wild-type group, the difference was smaller than that with the Δ*hylB* group.

**Figure 7:**
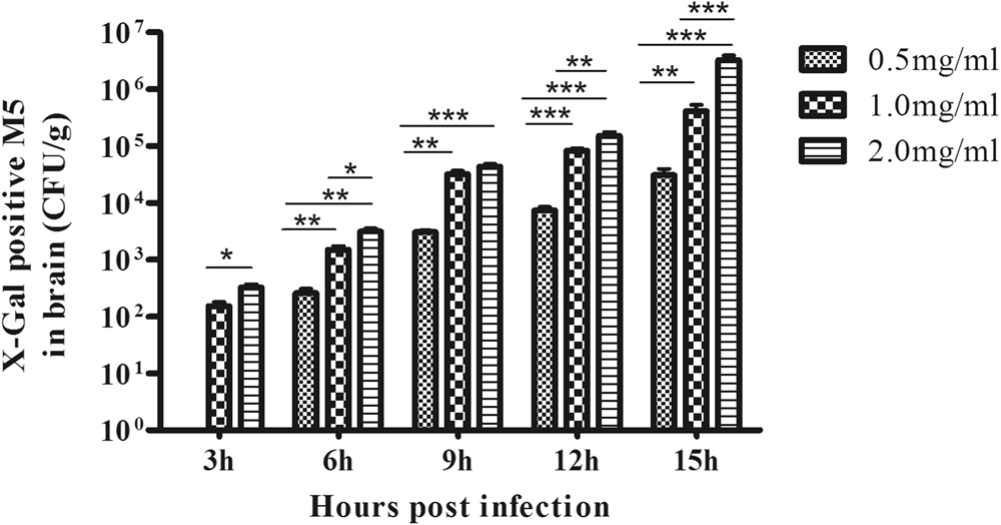
Evaluation of BBB opening in mice injected with different doses of hyaluronidase. Groups of four BALB/c mice were inoculated with 0.5, 1.0 or 2.0 mg/ml of HylB, and at different time points post-infection, *E. coli* M5 (2 × 10^8^ CFU) was injected intravenously. Five minutes later, the mice were killed, and the brains were removed for quantification of M5. The data are expressed as CFU per gram of brain. *P < 0.05, **P < 0.01 or ***P < 0.001 indicates significantly different bacterial loads between the two infection groups.

To further investigate the role of the *hylB* gene in BBB opening, the protein HylB, which is encoded by the *hylB* gene, was expressed and injected intravenously into the mice. An injection of 0.5 mg/mL HylB did not cause BBB opening at 3 h, as evidenced by the absence of *E. coli* M5 in brain, whereas M5 could enter and begin to accumulate in the brains of the 1.0 mg/mL and 2.0 mg/mL groups (Fig. 7). There was a dose-dependent increase in the degree of BBB opening at each time point.

## Discussion

Meningitis is the most common clinical syndrome of *S. agalactiae* infection. Bacterial penetration across the BBB and into the CNS is the first step in the development of meningitis^26^. Therefore, adequate BBB models need to be developed in order to characterize the properties of bacterial penetration into the CNS. The *in vitro* BBB model based on the culture of brain microvascular endothelial cells has been widely used to probe the potential role(s) of individual virulence determinants in the initial pathogenesis of CNS infection by *S. agalactiae*. For example, hBMEC has been used to determine the invasive roles of fibronectin binding protein A (SfbA)^27^, laminin-binding protein (Lmb)^28^ and the surface protein HvgA in GBS infection^29^. However, the *in vitro* model might not completely mimic the disease in animals or humans. It was reported that CovR-deficient GBS showed a decreased ability to invade the brain endothelium *in vitro*, but *in vivo*, this deletion mutant was more proficient in the induction of permeability and proinflammatory signaling pathways in the brain endothelium and in penetration of the BBB^30^. In contrast, a previous study on the major pilin subunit PilB reported that the *pilB* mutant was less virulent than its wild-type strain in the newborn mice model, whereas, *in vitro*, the mutant had the similar ability with the wild-type GBS in resistance to macrophage killing^31^. Therefore, the development of an *in vivo* model will be extremely helpful in the study of bacterial meningitis.

The mouse has been widely used for investigating *S. agalactiae* virulence^24,27,32^. In recent years, some researchers have developed mouse models by using intravenous injection of exogenous tracers in mice and subsequent detection of extravasate molecules in the brain tissues to measure BBB permeability^33,34^. The tracers provide convenient morphological evidence for BBB opening; however, Saunders *et al*.^35^ reported that the exogenous tracers are not a satisfactory tool for studying blood-brain barrier dysfunction. For example, Evans blue, one of the most commonly used markers, has toxic properties and thus the dye detected in brain might be due to toxic effects on cerebral endothelial or ependymal cells. Also, Evans blue detected in brain is likely to be a mixture of dye bound to plasma proteins, dye bound to brain tissue and free dye. Therefore, it is unreliable to estimate the size of BBB impairment using Evans blue. In this study, we established a model to assess the degree of BBB opening using *E. coli* M5, which has strong β-galactosidase activity, as an indicator. The bacterial strain is capable of producing blue colonies when cultured on medium containing X-Gal, and cannot permeate an intact BBB. Any entry of this bacterium into and spread within the brain is indicative of a leaky BBB. Therefore, the extent of BBB integrity can be quantitatively assessed through the monitoring of *E. coli* M5 and the measurement of the bacterial number in brain tissue. The similar method has been reported in *S. pneumonia* by Tsao *et al*.^26^ Nevertheless, the parameters and criteria may be variable due to different bacterial species. In our study, this model was optimized for use in *S. agalactiae*, and demonstrated to be a powerful method for analyzing the BBB opening.

Under physiological conditions, the BBB strictly regulates the entry of blood-borne substances into the brain. Brain inflammation can affect the permeability of the BBB directly via cytokine-mediated activation of metalloproteinases or tight junction disruption, or indirectly by promoting transmigration of leukocytes^36^. In *S. agalactiae*, hyaluronidase has been demonstrated to contribute to the bacterial invasion and the pathogenesis of meningitis in mice^24^. However, unlike the PilA and β-hemolysin/cytolysin which stimulate the release of pro-inflammatory cytokines^18,19^, hyaluronidase acts as an anti-inflammation factor instead. Our previous study demonstrated that compared to the wild-type *S. agalactiae*, the hyaluronidase- deficient mutant stimulated a significantly higher level of pro-inflammatory cytokines including IL-1β, IL-6 and TNF-α in macrophages, whereas its mortality to zebrafish was lower^24^. Afterwards, a probable reason for this phenomenon was illustrated by Kolar *et al*.^37^. They revealed that GBS evades host immunity by degrading hyaluronan (HA) which is a component of extracellular matrix nearly in all tissues. HA is commonly cleaved into small fragments by tissue hyaluronidase in response to tissue injury. These small HA fragments are inflammatory factors that ligate to Toll-like receptor (TLRs) to elicit inflammatory response and repair the damaged tissue. However, bacterial hyaluronidase degrades pro-inflammatory HA fragments to the major end product disaccharides. HA disaccharides bind to TLR2/4 to block signaling elicited by host HA fragments and other TLR2/4 ligands, thus preventing GBS ligands from activating pro-inflammatory signaling cascades. Therefore we assume that hyaluronidase contributes to GBS meningitis by anti-inflammation and evasion of host immune. However, our recent study showed that intravenous injection of a purified hyaluronidase, HylB, induced acute lung and brain injury^38^. This led to us to speculate that HylB might play important role in BBB permeability. In order to evaluate this speculation, we first investigated the role of HylB in disrupting the BBB integrity using this model established in this study. Compared with the wild-type *S. agalactiae*, the inactivation of *hylB* resulted in decreased BBB opening throughout the infection. Although the presence of *S. agalactiae* in brain indicates the BBB opening, the use of *E. coli* M5 as an indicator excludes the possibility that the differential BBB integrity may be caused by different proliferation abilities *in vivo* between the wild-type and *hylB* mutant strains.

To further determine whether HylB has a direct impact on BBB integrity, we intravenously injected the purified HylB protein into the mice. We found that the intravenous injection of HylB induced BBB opening in a dose-dependent manner. In the groups treated with 1 mg/mL and 2 mg/mL, the BBB was open 3 h post-infection, 3 h earlier than in the 0.5 mg/mL group, and the number of *E. coli* M5 increased with the time of infection. In considering the dose of HylB protein, we tested a treatment of 3 mg/mL HylB, but all the mice died 15 h post-infection. This finding indicated that HylB is one of the important virulence factors of *S. agalactiae*, which is in agreement with previous studies^24,38^. A similar role for hyaluronidase in inducing *pneumococcal* meningitis has also been reported by Zwijnenburg *et al*.^39^. The present investigation of HylB further demonstrates that using *E. coli* M5 as an indicator is an easy and reliable method for assessing BBB integrity and/or leakiness. In particular, the model could be more suitable to investigate the contribution of soluble bacterial virulence factors to BBB disruption.

In this study, we used a piscine strain of *S. agalactiae* with an extremely high virulence to BALB/c mice. It is not clear why this piscine strain is so virulent and what genetic relationship exists between fish and human isolates. Our previous study has made a comparative genomic analysis among 15 *S. agalactiae* strains of different origins, and found that the Chinese piscine isolates GD201008-001 and ZQ0910 are phylogenetically distinct from the Latin American piscine isolates SA20-06 and STIR-CD-17, but are closely related to the human strain A909^40^. Additionally, a published study reported that a GBS isolate from a clinical case of human neonatal meningitis caused disease and death in Nile tilapia^8^. In this regard, it may be of interest to further investigate the pathogenic mechanisms of meningitis caused by different origins of *S. agalactiae* strains. This model established here could be a potentially useful tool for the investigation. Nevertheless, it will be imperative to demonstrate that the *E. coli* tracer works with other GBS and mouse strains that are widely used in the meningitis model.

In summary, the present study developed a model that can quantify the degree of BBB opening caused by *S. agalactiae*, and used this model to demonstrate that hyaluronidase plays a direct role in BBB permeability.

## Methods

### Bacterial strains and growth conditions

*S. agalactiae* strain GD201008-001, β-hemolysin/cytolysin positive, which belongs to serotype Ia, MLST type ST-7, was isolated from farmed tilapia with meningoencephalitis in Guangdong Province, China, in 2010^40^. Its genome sequence has been deposited in the GenBank database under accession number CP003810. The *S. agalactiae hylB* deleted mutant strain Δ*hylB* and the complemented strain CΔ*hylB* were constructed in the previous study^24^. All of the bacterial strains were grown using either Todd-Hewitt broth (THB) or agar (THA) (Becton Dickinson, MD, USA) or sheep blood agar plates at 37 °C.

### Screening for a β- galactosidase-positive *E. coli* strain

To describe the time and degree of BBB opening, an indicator bacterial strain was needed. Inspired by blue-white selection, five strains of engineered *E. coli* (numbered from M1 to M5) were grown overnight in the dark at 37 °C in M63 basic medium (13.6% (w/v) KH_2_PO_4_, 0.4% (w/v) KOH, 0.2% (w/v) (NH_4_)_2_SO_4_, 0.1 mM FeSO_4_) containing 1 mM MgSO_4_, 0.2% (w/v) lactose, 0.002% (w/v) 5-bromo-4-chloro-3-indolyl-L-D- galactopyranoside (X-gal) and 0.002% (w/v) vitamin B_1_. The bacterial strains were screened for their ability to form blue colonies on M63 plates for use as an indicator in the study.

### Determination of *E. coli* M5 virulence in mice

BALB/c mice (24–26 g, aged 5–6 weeks) were bought from the Experimental Animal Center, Yangzhou University. The mice were divided into two groups with 10 mice for each group. The screened *E. coli* M5 were grown overnight in LB broth. A bacterial suspension of 50 µL was transferred into 5 mL LB and incubated at 37 °C to allow the cells to reach mid-log phase growth. When the bacteria reached an OD_600_ of 0.6, they were harvested by centrifugation at 5000 × *g* for 5 min. The cell pellets were washed twice with sterile phosphate-buffered saline (PBS) (pH 7.4) and re-suspended in PBS to a concentration of 2 × 10^10^ CFU/mL. One group of mice was injected intravenously with 100 μL of bacterial suspension, whereas the other was injected with 100 μL PBS and served as a control. The mice were observed until one week post infection.

### Kinetics of *E. coli* presence in blood of mice

As an indicator, *E. coli* M5 should be eliminated rapidly from the circulatory system. Based on five predetermined time points, BALB/c mice (24–26 g, aged 5–6 weeks) were divided into five groups with eight mice for each group. Mid-log phase bacteria were washed twice with PBS, followed by re-suspension in PBS and adjustment of the concentration to 2 × 10^9^ CFU/mL. For each time point, five mice were used as the experimental group and were injected intravenously with 100 μL of bacterial suspension, while another three were injected intravenously with 100 μL of PBS. Blood samples and brains were obtained aseptically at 3 min, 5 min, 10 min, 30 min and 60 min post infection. Blood samples of 100 μL were spread onto M63 plates. To avoid surface contamination, the organs were washed twice with PBS. Tissues were placed in 1 mL of PBS and homogenized with a biological sample homogenizer (BioPrep-24, Ningbo Hinotek Instrument Co Ltd, China). Then, 100 μL of homogenate that was either undiluted or diluted 10^−1^, 10^−2^ and 10^−3^ in PBS were plated on M63 plates. The M63 plates were incubated overnight at 37 °C. Colonies were counted and given as CFU/g for brain samples or CFU/mL for blood samples.

### Determination of the challenge concentration of *S. agalactiae*

Our previous study has shown that the bacterial strain GD201008–001 is highly virulent to BALB/c mice by intraperitoneal administration, with LD_50_ values of less than 10 CFU^25^. Here, we chose two different doses of *S. agalactiae*, 50 and 100 CFU (5- and 10-fold greater than the LD_50_), to find an applicable dose for this mouse model. BALB/c mice were divided into two groups with 16 mice for each group. One group received an intraperitoneal injection of 100 μL of 500 CFU/mL bacterial suspension, and the other received an injection of 1000 CFU/mL. In each group, four mice were sacrificed every three hours to aseptically collect the blood and brain. Homogenized brain tissues and blood were plated onto THB plates for bacterial cell counting to determine tissue colonization. The experiments were repeated at least three times to ensure reproducibility. The data are expressed as CFU/g or CFU/mL per mouse.

### Evaluation of BBB opening

BALB/c mice were divided into five groups with five mice for each group. The mice were infected with a predetermined dose of 100 CFU of the strain GD201008-001. Control mice were injected with sterile PBS. Then, 2 × 10^8^ CFU of the indicator *E. coli* M5 in 100 μL PBS was given intravenously at the specified time points (3 h, 6 h, 9 h and 12 h), and 5 min later, the mice were sacrificed. To detect the degree of BBB opening, the brains were removed aseptically and homogenized in PBS. The homogenate was serially diluted and spread onto THB or M63 agar plates, then incubated overnight at 37 °C. The organ CFU enumeration of *S. agalactiae* and *E. coli* M5 were determined and expressed as the mean and S.D. per mouse.

### Detection of BBB opening caused by *S. agalactiae* hyaluronidase

To investigate the effect of BBB opening caused by *S. agalactiae* hyaluronidase, we used the deficient mutant strain Δ*hylB* and the complemented strain CΔ*hylB* constructed in our previous study^24^. One hundred and twenty mice were divided into three groups with 40 mice for each group. Mid-log phase *S. agalactiae* and its derivatives were washed twice in PBS and re-suspended in PBS to 1 × 10^3^ CFU/mL. The concentration of *E. coli* M5 was adjusted to 2 × 10^9^ CFU/mL. Three groups of mice were infected with 100 µL of the wild-type *S. agalactiae*, Δ*hylB* or CΔ*hylB* by intraperitoneal injection. At 3 h, 6 h, 9 h, 12 h, and 15 h post infection, groups of four mice were killed, and the blood and brain tissues were collected for *S. agalactiae* quantification. Meanwhile, another four mice from each group were respectively inoculated with 100 µL of *E. coli* M5 by intravenous route at each time point as mentioned above. At 5 min post-inoculation with *E. coli*, the brains were aseptically removed and homogenized in PBS. The homogenates were serially diluted, spread onto THB plates for *S. agalactiae* counting or M63 plates for *E. coli* M5 counting and incubated overnight at 37 °C. The bacteria were counted and reported as CFU/g per mouse.

To further determine the role of the *hylB* gene in the pathogenesis of BBB opening caused by *S. agalactiae*, we expressed HylB with good enzymatic activity, as described in our previous study^38^. Sixty mice were divided into three groups with 20 mice in each group. The three groups of the mice were intravenously injected with 200 μL of HylB protein at a final concentration of 0.5 mg/mL, 1.0 mg/mL and 2.0 mg/mL, respectively. Another 20 control mice were injected with 200 μL of sterile PBS. Then, 100 μL of the *E. coli* M5 (2 × 10^9^ CFU/mL) was inoculated intravenously at intervals of 3 h, 6 h, 9 h, 12 h and 15 h post infection, and the brain tissues were sampled at 5 min post-infection with *E. coli*. The homogenates were serially diluted, spread onto M63 plates and incubated overnight at 37 °C for *E. coli* M5 counting. The bacteria were counted and expressed as CFU/g per mouse.

### Statistical analysis

Data were collected and analyzed using MS Excel 2010 and SPSS Statistics version 20.0 software. Multiple comparisons were performed by analysis of variance (ANOVA) followed by Turkey’s multiple-comparison test, with *P* < 0.05 indicating a statistically significant difference and *P* < 0.01 indicating a highly significant difference. The error bars presented in the figures represent the standard deviations of the means of multiple replicate experiments.

## Experimental procedures

### Ethics statement

All the animal experiments were carried out according to animal welfare standards and were approved by the Ethical Committee for Animal Experiments of Nanjing Agricultural University, China. All animal experiments complied with the guidelines of the Animal Welfare Council of China.
